# Commentary: Proinflammatory role of blister fluid-derived exosomes in bullous pemphigoid

**DOI:** 10.3389/fimmu.2020.01506

**Published:** 2020-07-16

**Authors:** Yangchun Liu, Li Li

**Affiliations:** Department of Dermatology, Peking Union Medical College Hospital, Peking Union Medical College and Chinese Academy of Medical Sciences, Beijing, China

**Keywords:** exosome, bullous pemphigoid, skin disorder, immunology, inflammation

## Introduction

Extracellular vesicles (EVs) are small membranous structures comprised of lipid bilayers. They can be secreted into several biofluids including urine, plasma, saliva, cerebrospinal fluid, synovial fluid, and breast milk (1). Exosomes and microparticles comprise the two major populations of EVs and differ from one another mainly by size and mechanism of generation. Exosomes are EVs ~30–100 nm in diameter. They are secreted by many immune and non-immune cell types including T cells, B cells, dendritic cells, and macrophages (2). They contain a wide array of biological materials including proteins, lipids, transcription factors, RNA, and DNA, and enable cell-to-cell communication by transporting their cargo and delivering it to target cells (3). Exosomes can mediate immune stimulation and suppression via antigen presentation, T cell activation, and anti-inflammatory activity (2). Exosome microRNAs can be used as putative diagnostic biomarkers to distinguish autoimmune diseases such as systemic lupus erythematous, rheumatoid arthritis, and dermatomyositis (4).

Bullous pemphigoid (BP) is the most common subepidermal autoimmune blistering disease of the skin (5). The non-collagenous 16A (NC16A) domain of BP180 and the C-terminal domain of BP230 are the major epitopes of BP. Both are hemidesmosome proteins, which are structural components of the hemidesmosomes that connect basal keratinocytes with the basement membrane zone (6). Binding of autoantibodies to hemidesmosome proteins causes degradation of the basement membrane zone and blister formation. This is accompanied by the activation of inflammatory cells (such as eosinophils, neutrophils, and mast cells) and cytokine production [such as interleukins and CC-motif chemokine ligands (CCLs)]. Fang et al. assessed the potential roles of exosomes in the inflammatory processes associated with BP using mass spectrometry. They detected the production of proinflammatory molecules, including interleukin (IL)-6, tumor necrosis factor (TNF)-α, and CXC-motif chemokine ligand (CXCL)-8, in cell-free supernatants of exosome-stimulated keratinocytes.

## Discussion

### The Mechanism of EV Interaction With Target Cells

Numerous studies have assessed the interaction of exosomes or EVs with target cells by fluorescence microscopy and flow cytometry. EVs interact with cells through several mechanisms (7). They secrete mediators that bind to receptors expressed on target cells, or may interact with target cells via direct membrane contact. This interaction causes the activation of different signal transduction pathways in target cells (1, 8, 9). Fang et al. (10) used fluorescence microscopy to demonstrate that exosomes derived from BP patient blister fluid were internalized by keratinocytes and subsequently activated ERK1/2 and STAT3 signaling.

### Inflammatory Events Involved in BP

Many inflammatory molecules have been postulated to play a role in the activity and intensity of BP. The concentrations of cytokines such as IL-1β, TNF-α, IL-5, IL-6, IL-8, IL-10, IL-15, IL-17, IL-23, and IL-31, and chemokines such as eotaxin-1 (CCL11) and eotaxin-3 (CCL26) are elevated in the sera and blister fluids of BP patients (11–15). Inflammatory proteins such as eosinophil cationic protein, major basic protein, and heat shock protein 90 also contribute to the BP inflammatory reaction (16, 17). Fang et al. (10) detected production of the proinflammatory molecules IL-6, TNF-α, and CXCL-8 following the incubation of blister fluid-derived exosomes with primary human keratinocytes. Other inflammatory molecules may be detected after the stimulation of keratinocytes with exosomes, which may help us further demonstrate the role of exosomes in BP inflammatory processes.

### The Potential Role of Exosomes in BP

Fang et al. (10) also conducted proteomic analyses of exosome contents and detected antibody fragments. Based on their findings, we speculate that exosomes may transport the pathogenic autoantibodies associated with BP including anti-BP180 and anti-BP230 antibodies. Following internalization, the autoantibodies carried by exosomes may be released to stimulate immune responses. Many studies have demonstrated that microparticles carry autoantigens, but few have cataloged the full immunological components of exosomes. The mass spectroscopy analyses conducted by Fang et al. did not detect antigen fragments targeted by BP-associated autoantibodies such as BP180 or BP230 (10). However, it remains to be determined whether exosomes derived from other body fluid, such as plasma and urine, contain autoantigens or related proteins.

## Conclusion

We commend the work of Fang et al. (10) for demonstrating the inflammatory role of blister fluid-derived exosomes in the pathogenesis of BP. Multiple studies have assessed the potential roles of exosomes in the pathogenesis of autoimmune diseases such as systemic lupus erythematosus, dermatomyositis, and rheumatoid arthritis. However, no study had investigated the inflammatory role of exosomes in BP pathogenesis. Continued research into the biology and functions of exosomes may facilitate the discovery of new diagnostic biomarkers and contribute to the development of new therapeutic agents for BP.

## Author Contributions

YL wrote the manuscript. LL edited the manuscript. All authors contributed to the article and approved the submitted version.

## Conflict of Interest

The authors declare that the research was conducted in the absence of any commercial or financial relationships that could be construed as a potential conflict of interest.
